# The effects of mobile phone use on motor variability patterns during gait

**DOI:** 10.1371/journal.pone.0267476

**Published:** 2022-04-21

**Authors:** Javad Sarvestan, Peyman Aghaie Ataabadi, Zdeněk Svoboda, Fatemeh Alaei, Ryan B. Graham

**Affiliations:** 1 Faculty of Physical Culture, Department of Natural Sciences in Kinanthropology, Palacky University Olomouc, Olomouc, Czech Republic; 2 Faculty of Physical Education and Sport Sciences, Department of Biomechanics and Sports Injuries, Kharazmi University, Tehran, Iran; 3 Faculty of Health Sciences, School of Human Kinetics, University of Ottawa, Ottawa, Canada; University of Illinois at Urbana-Champaign, UNITED STATES

## Abstract

Mobile phone use affects the dynamics of gait by impairing visual control of the surrounding environment and introducing additional cognitive demands. Although it has been shown that using a mobile phone alters whole-body dynamic stability, no clear information exists on its impacts on motor variability during gait. This study aimed at assessing the impacts of various types of mobile phone use on motor variability during gait; quantified using the short- and long-term Lyapunov Exponent (λ_S_ and λ_L_) of lower limb joint angles and muscle activation patterns, as well as the centre of mass position. Fourteen females and Fifteen males (27.72 ± 4.61 years, body mass: 70.24 ± 14.13 Kg, height: 173.31 ± 10.97 cm) walked on a treadmill under six conditions: normal walking, normal walking in low-light, walking while looking at the phone, walking while looking at the phone in low-light, walking and talking on the phone, and walking and listening to music. Variability of the hip (p λ_S_ = .015, λ_L_ = .043) and pelvis (p λ_S_ = .039, λ_L_ = .017) joint sagittal angles significantly increased when the participants walked and looked at the phone, either in normal or in low-light conditions. No significant difference was observed in the variability of the centre of mass position and muscle activation patterns. When individuals walk and look at the phone screen, the hip and knee joints are constantly trying to adopt a new angle to regulate and maintain gait stability, which might put an additional strain on the neuromuscular system. To this end, it is recommended not to look at the mobile phone screen while walking, particularly in public places with higher risks of falls.

## Introduction

Smartphone use is on the rise all over the world, making it the most important item in people’s daily life [1]. In recent years, individuals rarely just walk, but rather teenagers and adults constantly interact with their smartphones during walking (e.g., listening to music, looking at the screen while texting or browsing, and talking on the phone) [2]. Walking while doing another task on the phone (i.e., dual-tasking) necessitates higher cognitive, neuromotor, memory and physical capabilities [3,4]. Smartphone usage requires continuous focus on the screen (flexed head and neck positions) combined with high levels of manual dexterity [3]. This means that using a mobile phone while walking requires high levels of working memory and executive function and limits visual control and awareness of their surroundings, which increases the possibilities of colliding with obstacles and falling [5]. Compared to walking and talking on a cell phone, individuals are more prone to falling while walking and texting, which could be due to limited visual inputs [6,7].

Gait performance is not a fully automated function and needs constant attention and adaptation to the surrounding environment [8–10]. As a result, it has been hypothesized that mobile phone use could impair individuals’ dynamic stability during gait [11]. Researchers have analysed whole-body local dynamic stability (LDS), using nonlinear methods such as short- and long-term local Lyapunov Exponent (LyE, which is represented by λ), to analyse the impacts of mobile phone use on gait performance [3,6,11]. LyE is a principal variable that portrays the chaos levels in a system [12]. Greater values of LyE represent larger variability in a system [12]. LyE has been validated as a reliable measure for the prediction of whole-body LDS during walking and using the mobile phone [13], where increased λ_S_ and λ_L_ values indicate an increase in movement variability or decrease in the LDS of a system [13]. Besides, LyE has lately been employed as a measure of variability in joint motions and muscle activity patterns in order to precisely analyse individual segments as a whole [14].

Although both talking and texting disrupt arm-swing, it seems that visual impairment has a larger effect on gait alterations. Walking and talking or texting on a phone significantly decreases whole-body LDS in the mediolateral (frontal) plane, where the changes from texting are more significant than talking [3]. To support this, no differences were observed between the LDS of normal walking and walking and talking on the phone among young and older adults [11]. However, the centre of mass (COM) trajectory (representing the whole-body LDS) was the sole parameter studied in previous research investigations on the effects of cell phone use on the LDS of gait, while the impact of mobile phone use on the LDS of the lower limb joints and muscles acting around them is still unclear.

The neuromuscular system generates and manages forces to drive kinematics and maintain its stability [15]. Among the scarce studies conducted on the impacts of mobile phone use on gait performance, it has been evidenced that walking and texting significantly increases co-contraction between the ankle agonist and antagonists muscles at approximately mid-stance [2], which highlights an augmented need for ankle stabilization. Additionally, erector spinae muscle activity increased considerably as a result of browsing and texting [16]. Nonetheless, no research study has investigated the impacts of mobile phone use on lower limb muscle activation variability during walking and using the mobile phone. Muscular activity LDS was formerly analysed in lifting tasks, trunk movements and pedalling [15,17,18], but not in gait performance. Understanding how muscle activation patterning (generated force) is altered, as well as how this relates to gait control is critical, particularly when individuals use a mobile phone during walking.

Maintaining stability during walking is constantly controlled by the neuromuscular systems, and requires high levels of attention to adapt to the surrounding environment, particularly when individuals use their smartphones. Whole-body LDS has been shown to be altered during walking and using a mobile phone; however, precise analysis is required to analyze the impacts of mobile phone use on the LDS of lower limb joint angles and muscular activity. To this end, this study aimed to investigate the impacts of various types of mobile phone use on motor variability during gait; quantified using the short- and long-term local divergence exponents (λ_S_ and λ_L_) of lower limb joint angles and muscle activation patterns, as well as the centre of mass position. It was hypothesized that any condition involving looking at the mobile phone screen during walking would result in higher λ values of lower limb joint angles and muscle activities, and consequently increase the COM λ measures (less stability).

## Materials and methods

### Study design

This study adopted a cross-sectional study design where the experimental conditions were: a) normal walking (NW); b) walking while looking at the phone (searching through social media using one hand- WLP); c) walking in low-light condition (WLL); d) walking in low-light condition while looking at the phone using one hand (WLLLP); e) walking while listening to the music (using a headset—WM), and f) walking while talking through the phone (keeping the phone near the ear by one hand- WTP). The dependent variables were the short- (λ_S_) and long-term (λ_L_) local divergence exponents of the COM trajectories; pelvis, hip, knee and ankle angles in three dimensions; and muscular activities.

### Participants

Twenty-nine healthy adults (14 females, 15 males, age: 27.72 ± 4.61 years, body mass: 70.24 ± 14.13 Kg, height: 173.31 ± 10.97 cm, BMI: 23.24±3.32) volunteered to participate in this study. A priori power analysis, using G*Power 3.1.9, indicated that a sample size of 28 would be sufficient with power (1 - β) of 0.95, an α of 0.05, and an effect size of 0.25 [19,20]. Participants had no history of severe injuries or surgery in the lower extremities, including muscle or ligament rupture, joint laxation and bone fracture, within the last 12 months of the measurement procedure. The entire test protocol was thoroughly explained (verbally and written) to the participants and they signed the written informed consent prior to the measurement procedure. The study was conducted according to the guidelines of the Declaration of Helsinki and approved by the Institutional Review Board of Faculty of Physical Culture, Palacky University Olomouc [ethic code: 8/2021].

### Experimental procedure

The dominant leg of each individual was identified prior to the warm-up protocol using a ball-kicking test [21]. Following that, participants walked eight times through a 10-metre pathway at their desired pace. Their walking pace was then determined by dividing the distance travelled by the time spent walking in each trial. The participants were then instructed to walk at the same speed on the treadmill in order to adapt their walking performance.

Thereafter, 6 wireless surface electrodes were placed in the middle of the bulky part of the Gluteus Medius (GM), Rectus Femoris (RF), Vastus Medialis (VM), Biceps Femoris (BF), Medial Gastrocnemius (MG), Tibialis Anterior (TA) (Trigno™ Wireless Systems; Delsys Inc, Natick, MA) [22]. The maximum voluntary isometric contraction (MVIC) of these muscles was recorded in two series of 5-second contractions. We used the functional resistance test to ensure that the target muscle was detected to reduce the effects of crosstalk [23]. Thereafter, 15 retroreflective 14mm-diameter passive markers were attached to the C_7_ and T_10_ vertebrae, clavicle, sternum, scapula, anterior superior iliac spines, posterior superior iliac spines, mid-thighs, lateral femoral epicondyle, mid-tibias, lateral malleoli, first metatarsal and heels of the dominant leg by an expert researcher using the PlugInGait model. One marker was also placed on the midway between posterior superior iliac spines in order to track the COM’s 3-planar motions [24].

Prior to test execution, participants were provided with the opportunity to familiarize themselves with treadmill walking at their preferred speed over the ground. The participants later walked on the treadmill under the following 6 circumstances:

- NW: the participants walked on the treadmill like they would on a regular basis.
- WLP: the participants walked on the treadmill while they looked and worked with their mobile phones using one hand. They were asked to choose the most frequently used application (game, social media, texting, etc.) or webpage during walking. Given that they worked with the most frequently used application (to simulate real-life circumstances), we did not restrict them to use a particular application. Nevertheless, Instagram was the most frequently used application among the participants (n = 13, 45%). Their eye-screen contact was checked by the researcher throughout walking.
- WLL: the participants walked on the treadmill as they would on a regular basis in a low-light condition (Dimming the lights -average 50 lux- in the room maintained low-light conditions) [25].
- WLLLP: in the WLL condition, the situation was identical to that of WLP.
- WM: the participants walked on the treadmill while they listened to their favourite music using a headset. Pop (n = 12, 41%) and rock (n = 7, 24%) were the most frequent listened to genres. Jazz, metal, country and electro were the other genres.
- WTP: the participants walked on the treadmill while they had a real voice call in their official language. The conversation was about general daily topics, including daily activities, sport, study, job, travelling, etc. However, the questions were ordered from easy to hard so that the participants cognitively dealt with finding proper answers. Although different people were in charge of phone calls (to speak in the participants’ official language), the defined questions were identical so that all the participants answered similar questions. Throughout the call, they were asked to keep the phone next to their ears by their hands.

In this study, we adopted the low-light condition only for the normal and WLP condition to observe the impacts of different visual control on the walking performance when the visual input decreases due to the low-light condition. The treadmill screen was obscured throughout all situations so that the participants’ concentration was not distracted. The 3D kinematic data of segments and joints on the markered dominant side were recorded using 6 optoelectronic cameras (Vicon^®^ VCAM motion capture system, Oxford Metrics, Oxford, United Kingdom) at the sampling frequency of 100Hz). Each test lasted 3 minutes, with 2 minutes of rest between conditions, and each subject performed the conditions in a randomized order [3].

### Data analysis

The Euler angles between the foot, shank, pelvis and trunk were set and used to calculate the ankle, knee, hip and pelvis angles in 3 dimensions [26]. In this study, for each walking condition, we trimmed the data to 150 strides and then normalized the data to 15000 time points to maintain a consistent number of strides and data points across all participants and experimental conditions [27]. For the non-linear analyses, LDS was calculated adopting λ_S_ and λ_L_ of the COM trajectories, the pelvis, hip, knee and ankle joints angles and the lower limb muscular activities throughout all gait cycles under each condition. Rosenstein (1993) algorithm was used for LDS analysis [12,28]. For EMG data, prior to the LDS calculations, the raw EMG data were band-pass filtered between 30 and 450Hz using a 4^th^ order Butterworth recursive filter. Thereafter, to generate a linear envelope for each of the six muscles during each gait cycle, these data were full-wave rectified and low-pass filtered using a 2^nd^ order Butterworth filter (9Hz cutoff frequency) and then divided by the MVIC values to calculate the activity percentage for each gait cycle [29].

**Fig 1:**
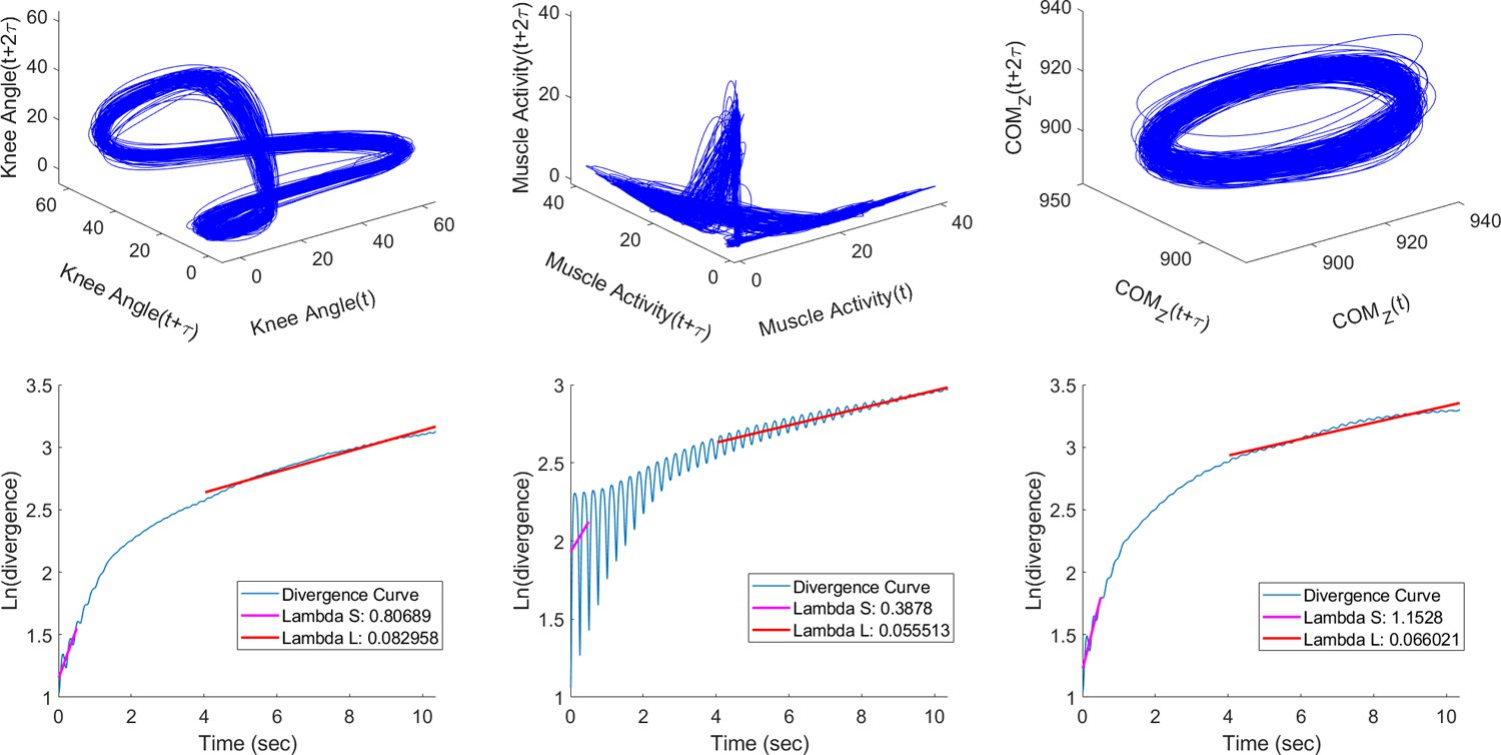
Schematic representation of the estimation of the maximum finite-time Lyapunov exponent for the knee sagittal plane (left), rectus femoris activities (centre) and COM trajectories (right). The 3D reconstruction of the phase-space and the expanded view of the reconstructed phase-space (up), and the average logarithmic rate of divergence for λ_S_ and λ_L_ (down).

For estimation of the time delays, the first minimum of the average mutual information function was computed [30]. Since each participant had a different pace, we calculated the time delay and embedding dimension for each participant based on their normal walking condition and applied this to all other conditions. The median embedding time delay for the entire tests was 23 (ranging from 18 to 28) for the kinematics data and 29 (ranging from 23 to 35) for the muscle activities. Thereafter, the d_E_ was computed from the global false nearest neighbours analysis and the d_E_ of 5 to 8 (for kinematics) and 7 to 10 (for muscle activities) was chosen in further calculations [31,32]. Then, the phase-spaces were reconstructed from the COM, all sagittal, frontal and horizontal plane angles of each joint, and EMG using the delay-coordinate embedding methods [33], as follows:

y(t)=[r(t),r(t+τ),r(t+2τ),…,r(t+(n−1)τ)] (1)

where the state vector is represented by y(t), x(t) is the original time-series, the constant time delay is presented as τ and n is the number of reconstruction dimension. We determined the Euclidian distances between neighbouring trajectories as a function of time after the phase-space construction process. Then, the mean of the entire pairs of nearest neighbours was used to calculate the average logarithmic rate of divergence, using the following equation:

$$y(i)=\frac{1}{\Delta t}(ln\;dj(i))$$ (2)

where d_j_(i) stands for the Euclidean distance between the pairs of nearest neighbours at i discrete time steps. Then, the calculated slope of the resulting divergence curves was considered as an estimation of the maximum finite-time LyE [34]. λ_S_ was calculated from the slope of 0 to 0.5 strides, while the λ_L_ was calculated from the slope of 4 to 10 intervals (Fig 1) [27].

### Statistical analysis

The normality of data distribution was checked using the Shapiro-Wilk test. The repeated-measures ANOVA was used to determine significant differences between the λ_S_ and λ_L_ of the COM trajectories, the pelvis, hip, knee and ankle angles, and the muscular activities at six different walking conditions. The Bonferroni post-hoc test was employed to identify the significant differences between the λ_S_ and λ_L_ of every two different conditions. The significance level was set at α = .05, and MATLAB software (version 2020b; MathWorks, Inc, Natick, MA) was employed to perform the entire data and statistical analyses described in the data and statistical analysis sections.

## Results

The Shapiro-Wilk statistical test confirmed the normality of data distribution. The calculated walking speed was 4.46±.51 km.h^-1^ for the group of participants. Descriptive measures of λ_S_ and λ_L_ for the ankle, knee, hip and pelvis angles, and the COM and their differences in various walking conditions in sagittal, frontal and horizontal planes are presented in Table 1.

**Table 1:** Descriptive measures of λ_S_ and λ_L_ for the ankle, knee, hip and pelvis angles, and the COM and their differences in various walking conditions in sagittal, frontal and horizontal planes.

			NW	WLP	WLL	WLLLP	WM	WTP	*F*	*p*
**Ankle**	**Sagittal**	**λ**_**S**_	1.167(.405)	1.191(.296)	1.091(.289)	1.103(.239)	1.152(.356)	1.112(.309)	.772	.572
		**λ**_**L**_	.006(.004)	.006(.005)	.007(.005)	.006(.004)	.006(.006)	.006(.004)	.222	.953
	**Frontal**	**λ**_**S**_	1.309(.362)	1.286(.329)	1.254(.276)	1.290(.310)	1.259(.306)	1.268(.283)	.168	.974
		**λ**_**L**_	.006(.004)	.006(.003)	.006(.004)	.006(.005)	.005(.006)	.005(.004)	.436	.823
	**Transverse**	**λ**_**S**_	1.257(.392)	1.227(.338)	1.204(.331)	1.278(.375)	1.261(.328)	1.223(.369)	.347	.884
		**λ**_**L**_	.006(.003)	.006(.004)	.006(.004)	.005(.005)	.006(.003)	.006(.005)	.083	.995
**Knee**	**Sagittal**	**λ**_**S**_	.991(.423)	1.014(.0504)	1.016(.505)	1.007(.503)	1.032(.509)	1.039(.524)	.127	.986
		**λ**_**L**_	.008(.006)	.009(.006)	.008(.006)	.009(.008)	.009(.008)	.009(.007)	.097	.992
	**Frontal**	**λ**_**S**_	1.051(.403)	1.097(.408)	1.106(.349)	1.119(.346)	1.074(.423)	1.112(.333)	.289	.919
		**λ**_**L**_	.007(.005)	.008(.006)	.008(.006)	.008(.008)	.008(.007)	.007(.005)	.107	.991
	**Transverse**	**λ**_**S**_	1.177(.315)	1.194(.319)	1.218(.397)	1.190(.363)	1.193(.414)	1.218(.357)	.142	.982
		**λ**_**L**_	.005(.004)	.006(.004)	.006(.004)	.006(.003)	.005(.004)	.006(.004)	.191	.966
**Hip**	**Sagittal**	**λ**_**S**_	.720 (.147)^a^^,^^b^	.807(.247)	.725(.137)^a^^,^^b^	.854(.188)	.721(.118)^a^^,^^b^	.736(.136)^a^^,^^b^	8.588	**.007** ‡
		**λ**_**L**_	.007(.005)^a^^,^^b^	.012(.008)	.009(.007)^a^^,^^b^	.013(.009)	.009(.007)^a^^,^^b^	.008(.006)^a^^,^^b^	2.765	**.021†**
	**Frontal**	**λ**_**S**_	.876(.389)	.871(.372)	.903(.338)	.899(.370)	.872(.392)	.929(.318)	.235	.947
		**λ**_**L**_	.005(.005)	.006(.007)	.006(.004)	.007(.007)	.006(.006)	.007(.006)	.215	.956
	**Transverse**	**λ**_**S**_	1.088(.307)	1.030(.375)	1.045(.277)	1.087(.291)	1.092(.319)	1.014(.339)	.484	.788
		**λ**_**L**_	.006(.008)	.006(.007)	.006(.005)	.007(.006)	.007(.006)	.007(.005)	.139	.983
**Pelvis**	**Sagittal**	**λ**_**S**_	.843(.363)^a^^,^^b^^,^^c^	.974(.389)^a^	.893(.422)^a^^,^^b^	1.137(.489)	.889(.339)^a^^,^^b^	.942(.418)^a^	2.666	**.025†**
		**λ**_**L**_	.006(.004)^a^^,^^b^	.012(.013)	.007(.006)^a^^,^^b^	.013(.018)	.008(.004)^a^^,^^b^	.008(.008)^a^^,^^b^	2.356	**.043†**
	**Frontal**	**λ**_**S**_	1.167(.394)	1.188(.385)	1.156(.280)	1.208(.324)	1.180(.277)	1.116(.234)	.329	.825
		**λ**_**L**_	.007(.007)	.008(.011)	.007(.006)	.007(.006)	.008(.008)	.008(.009)	.130	.985
	**Transverse**	**λ**_**S**_	.741(.212)	.749(.254)	.766(.175)	.733(.262)	.754(.204)	.799(.208)	.449	.814
		**λ**_**L**_	.006(.005)	.008(.005)	.007(.006)	.007(.005)	.006(.005)	.007(.005)	.810	.544
**COM**	**Sagittal**	**λ**_**S**_	1.015(.249)	.986(.243)	.961(.228)	.958(.216)	1.028(.207)	1.033(.241)	.758	.581
		**λ**_**L**_	.019(.012)	.020(.015)	.015(.012)	.016(.015)	.018(.010)	.019(.013)	.705	.621
	**Frontal**	**λ**_**S**_	.866(.274)	.972(.225)	.944(.339)	.986(.243)	.980(.291)	.868(.236)	1.279	.276
		**λ**_**L**_	.022(.018)	.028(.022)	.027(.017)	.024(.019)	.023(.020)	.020(.015)	.878	.497
	**Transverse**	**λ**_**S**_	1.336(.251)	1.331(.280)	1.323(.240)	1.363(.256)	1.311(.290)	1.284(.248)	.526	.757
		**λ**_**L**_	.008(.009)	.007(.005)	.007(.006)	.008(.006)	.008(.006)	.007(.006)	.417	.836

† Significantly different at α<0.05.

‡ Significantly different at α<0.01.

a significantly different with WLP at p < .001.

b significantly different with WLLLP at p < .001.

c significantly different with WTP at p < .001.

Note: Given the high number of conditions, we reported the differences only in one condition to prevent duplication. For instance, if the λ values for WN and WLP were significantly different, we reported that in the WN condition.

There was a significant main effect of the eye-screen contact on the variability of the hip joint angles in the sagittal plane, where the λ_S_ (F = 8.588, p = .007) and λ_L_ (F = 2.765, p = .021) were significantly higher in WLP and WLLLP in comparison with other walking conditions. Bonferroni post hoc test depicted significantly lower λ_S_ and λ_L_ values for WN, WLL, WM, WTP conditions in comparison with the WLP and WLLLP (*p* < .001). The repeated measures ANOVA test failed to show differences between the λ_S_ and λ_L_ of the hip angles in frontal and horizontal planes among all walking conditions.

As for the pelvis angles in the sagittal plane, the ANOVA test revealed a significant main effect for λ_S_ (*F* = 2.666, *p* = .025) and λ_L_ (*F* = 2.356, *p* = .043). Post hoc tests highlighted significantly greater λ_S_ measures for the pelvis sagittal angles during WLP, WLLLP and WTP (*p* < .001) in comparison with NW and WLL; while the pelvis λ_S_ measures were significantly lower in WLP, WM and WTP (p < .001) in comparison with WLLP. Regarding the λ_L_ measures, the Bonferroni test portrayed significantly greater values in WLLLP (p < .001) compared to all other walking conditions.

No considerable difference was observed in the λ_S_ and λ_L_ of the pelvis frontal and horizontal angles. No significant difference was observed between the ankle and the knee angles and the COM trajectories of the entire walking conditions in 3 dimensions. Table 2 depicts the descriptive measures of λ_S_ and λ_L_ for the RF, VM, GA, TA, BF, and GM and their differences in various walking conditions in sagittal, frontal and horizontal planes. As results portrayed, no significant difference was observed between the λ_S_ and λ_L_ of the RF, VM, GA, TA, BF, and GM during different walking circumstances.

**Table 2:** Descriptive measures of λ_S_ and λ_L_ for the RF, VM, GA, TA, BF, and GM and their differences in various walking conditions in sagittal, frontal and horizontal planes.

		NW	WLP	WLL	WLLLP	WM	WTP	F	p
**RF**	**λ**_**S**_	.805(.325)	.862(.351)	.868(.375)	.886(.473)	.938(.206)	.848(.187)	.576	.718
	**λ**_**L**_	.006(.004)	.007(.005)	.005(.004)	.007(.005)	.006(.006)	.005(.003)	1.033	.403
**VM**	**λ**_**S**_	.828(.338)	.839(.378)	.805(.351)	.866(.283)	.882(.171)	.950(.274)	.003	.958
	**λ**_**L**_	.006(.005)	.007(.006)	.006(.006)	.007(.006)	.006(.005)	.005(.004)	.794	.556
**GA**	**λ**_**S**_	.863(.318)	.885(.397)	.905(.395)	.936(.322)	.948(.228)	.857(.300)	.836	.368
	**λ**_**L**_	.007(.004)	.006(.005)	.006(.005)	.006(.005)	.004(.004)	.005(.004)	.213	.648
**TA**	**λ**_**S**_	.852(.356)	.821(.326)	.856(.391)	.910(.356)	.916(.227)	.895(.337)	.375	.865
	**λ**_**L**_	.005(.005)	.005(.004)	.005(.004)	.006(.005)	.004(.004)	.005(.005)	.580	.715
**BF**	**λ**_**S**_	.846(.446)	.860(.422)	.826(.359)	.837(.323)	.948(.202)	.894(.368)	.480	.790
	**λ**_**L**_	.007(.009)	.007(.005)	.006(.005)	.007(.004)	.007(.005)	.006(.006)	.296	.915
**GM**	**λ**_**S**_	.942(.352)	.884(.323)	.947(.444)	.924(.472)	.970(.262)	.878(.371)	.298	.913
	**λ**_**L**_	.006(.004)	.005(.005)	.007(.004)	.007(.006)	.005(.003)	.006(.006)	1.172	.326

RF = Rectus Femoris, VM = Vastus Medialis, GA = Gastrocnemious, TA = Tibialis Anterior, BF = Biceps Femoris, GM = Gluteus Medius.

## Discussion

This study aimed at assessing the impacts of various types of mobile phone use on motor variability during gait; quantified using the short- and long-term Lyapunov Exponent (λ_S_ and λ_L_) of lower limb joint angles and muscle activation patterns, as well as the centre of mass position. For this aim, adopting a cross-sectional study design, the gait cycle of 34 healthy adults was analysed during NW, WLP, WLL, WLP, WM, and WTP. Our hypothesis was partially supported by our findings because the hip angles in the sagittal plane demonstrated significantly higher values of λ_S_ and λ_L_ in WLP and WLLLP when compared to the other walking conditions. Furthermore, considerably higher values of λ_S_ and λ_L_ were observed in pelvis angles in WLLLP compared to the rest of walking conditions, while no significant difference was observed between the λ_S_ and λ_L_ values of COM, ankle and knee joint angles, and lower limb muscular activity.

It is generally hypothesized that reduced variability is linked with the increased stability of a system [11]. Although the above-mentioned hypothesis might be true when we analyse the system as a whole, the outcomes of this study revealed that we cannot generalize it to the sub-systems [35]. According to Kao, Higginson (11), errors in neuromusculator control could result in impairments in task performance or mechanical instability, which was highlighted as “bad variability”. Nevertheless, not all motor variabilities should be categorised as bad variability because they (good variabilities) might represent the flexibility of the central nervous system to adopt new strategies to control unpredicted circumstances and enhance mechanical stability [11]. Therefore, variability could be interpreted as bad variability when the whole-body stability is concerned, while a good variability could be predicated to those sub-systems that constantly change to maintain the whole system balanced. No significant difference between the λ values of the COM trajectories highlights that any type of mobile phone use (under the same walking speeds) has not decreased the whole-body LDS. However, given that former studies portrayed different outcomes [3,6], the question regarding the reasons for the lack of differences may arise. The main reason for these differences might be linked to the methodological approaches. Crowley, Vuillerme (6) analysed the impacts of texting on LDS while participants walked over the ground. As previous studies pointed out, cell phone use decrease walking speed [2,36], which could alter the whole-structure LDS because the gait patterns were changed in comparison with normal walking with preferred speed [27]. Moreover, Magnani, Lehnen (3) adopted a similar walking speed (4 km.h^-1^) for all participants, which could alter gait patterns since the participants walked at a different speed than their preferred speed. In this study, we adopted the same strategy (used preferred speed over the ground for treadmill walking) adopted by Kao, Higginson (11), and interestingly, their results support the findings of this study. Nonetheless, they did not monitor the lower limb joint strategies used to keep the whole body balanced.

A plethora of research studies documented that visual impairments could highly alter gait patterns [5,7]. Our results, in accordance with formerly-mentioned studies, illustrated that the pelvis and the hip angle variability significantly increased in the sagittal plane when the participants were looking at the screen (either in normal or in low-light condition). Thus, the notion of good variability, in this study, is predicated on the pelvis and hip joints, where they constantly adopted new angles at each stride to regulate the whole-body structure when the visual control was decreased. Besides, given that increment in the ankle (talocrural joint) angle variability could result in a less stable joint, its variability decreased to provide the structure with a firm basement to maintain dynamic balance at each step. Nevertheless, since this decrement was insignificant, it seems that the neuromuscular system placed a priority over the hip and pelvis joint to regulate the control between upper- and lower limbs to maintain total body stability.

More recently, Crowley, Vuillerme (6) hypothesized that visual impairment is not the main reason for the lower LDS values throughout walking and texting, but cognitive impairment (being cognitively dealt with the concept of texting) could attenuate it. On the contrary, our result illustrated that cognitive impairment is not the main contributor to the lower LDS if individuals have proper visual control over the environment. As could be seen, talking on the cellphone had no impact on the whole-body LDS and joints variability, while participants had to deeply think about the questions in their call. Furthermore, it could be observed that the ipsilateral arm-swing cannot solely deteriorate gait dynamic stability, since the participants held the phone next to their earls, and indeed, their arm-swing was interrupted. This outcome was supported by the study conducted by Punt and Bruijn [37].

This research investigation was the first study to analyse mobile phone use in night-simulated conditions. We hypothesised that less visual environmental input (integrated loss of central and peripheral vision) could considerably decrease dynamic stability during gait [38]. However, no significant difference was observed as the low-light condition was applied. To this end, it could be claimed that the central vision impairment (when subjects’ gaze was on the screen) had the most significant impact on the dynamic stability of the gait. Nevertheless, Graci, Elliott (38) brought up that peripheral vision is mainly in charge of the proprioceptive information (i.e., obstacles) to fine-tune the gait stability. Hence, given that our study was conducted on a treadmill (no-obstacle condition), the lack of differences between the low-light conditions and normal conditions seems logical. Thus, we suggest further research studies to investigate night-simulated walking condition on natural surfaces (grass, asphalt, etc.).

In contrast with our hypotheses, mobile phone use had no impact on the LDS of lower limb muscular activities. Our results were in line with the study conducted by Agostini, Fermo (2), where no significant difference between the rectus femoris, lateral hamstring, gastrocnemius and tibialis anterior muscle activity during mobile phone use. This illustrates that any type of mobile phone use, regardless of visual or ipsilateral arm-swing impairments, has no significant effect on the patterning of lower limb muscle activity during gait. Although we did not analyze the lower limb muscle activity percentages, lack of variability in muscle activity patterning could portray that the central nervous system autonomically controlled muscular activity patterning during gait, regardless of the constraints. Furthermore, although a significant difference between the hip and pelvis sagittal angle variability was observed, it seems the hip and pelvis flexors/extensors underwent no big effort to regulate this change. It could be due to the size of these muscles and the simplicity of the task (normal gait performance).

## Limitations

One of the limitations of this study was a lack of control over the working-with-phone condition, where individuals searched social media, texted or played online games. Nevertheless, given that we asked them to use the most frequent application used, we tried to establish a real-life circumstance for them. The same condition was applied to the listening to music condition, where the participants listened to their favourite music, regardless of the music type.

On the other hand, given that we aimed to investigate the variability of individual joint angles, we were not able to ask the participants to walk over the ground in a straight path for 3 minutes. To this effect, the outcomes of this study must be cautiously applied to the overground walking conditions. Nevertheless, since the overground walking velocity is higher during NW, compared with other types of mobile phone use, a fixed walking speed (similar to what individuals walk in their normal walking without external perturbations) on a treadmill could impose what is a sub-optimal walking speed for that mobile phone use condition [6].

## Conclusion

Mobile phone use did not affect whole-body stability during gait at preferred speed. According to the outcomes of this study, it seems that the central nervous system constantly regulates the hip and the pelvis joints angle to maintain the whole-body stability during gait when individuals look at their cell phones. Our findings suggest that not every type of mobile phone use could attenuate the whole-body stability during gait, but those activities that deal with visual control over the screen of the mobile phone could highly alter the patterning of the gait cycle in the hip and pelvis joints. Thus, a long-term hip and pelvis variability during walking, to constantly regulate the purturbations, might result in excessive lumbo-pelvic-hip muscles activation and decrease gait economy. Besides, although looking at mobile phone, in daily life, might not end up in falling when individuals walk in a non-obstacle conditions, it could highly result in a fall when obstacles arise as the visual control decreases. Furthermore, mobile phone use has no impact on the patterning of lower limb muscular activities during gait, which may portray an automatized control by the central nervous system. Nonetheless, we cannot say if muscle activities have risen or decreased; all we can say is that variability in muscle activity patterns has stayed constant under any circumstances.

## Supporting information

S1 Dataset Plos one dataset. (XLSX)
